# Impedance of nanometer thickness ferromagnetic Co_40_Fe_40_B_20 _films

**DOI:** 10.1186/1556-276X-6-468

**Published:** 2011-07-23

**Authors:** Shien Uang Jen, Tzu Yang Chou, Chi Kuen Lo

**Affiliations:** 1 Institute of Physics, Academia Sinica, Taipei, Taiwan, 11529, Republic of China; 2 Physics Dept, National Taiwan Normal University, Taipei, Taiwan, 11677, Republic of China

**Keywords:** spin-wave resonance, impedance, magnetic films

## Abstract

Nanocrystalline Co_40_Fe_40_B_20 _films, with film thickness *t*_f _= 100 nm, were deposited on glass substrates by the magnetron sputtering method at room temperature. During the film deposition period, a dc magnetic field, *h *= 40 Oe, was applied to introduce an easy axis for each film sample: one with *h*||*L *and the other with *h*||*w*, where *L *and *w *are the length and width of the film. Ferromagnetic resonance (FMR), ultrahigh frequency impedance (IM), dc electrical resistivity (*ρ*), and magnetic hysteresis loops (MHL) of these films were studied. From the MHL and *r *measurements, we obtain saturation magnetization 4*πM_s _*= 15.5 kG, anisotropy field *H*_k _= 0.031 kG, and *r *= 168 mW.cm. From FMR, we can determine the Kittel mode ferromagnetic resonance (FMR-K) frequency *f*_FMRK _= 1,963 MHz. In the *h*||*L *case, IM spectra show the quasi-Kittel-mode ferromagnetic resonance (QFMR-K) at *f*_0 _and the Walker-mode ferromagnetic resonance (FMR-W) at *f_n_*, where *n *= 1, 2, 3, and 4. In the *h*||*w *case, IM spectra show QFMR-K at *F*_0 _and FMR-W at *F_n_*. We find that *f*_0 _and *F*_0 _are shifted from *f*_FMRK_, respectively, and *f_n _*= *F_n_*. The in-plane spin-wave resonances are responsible for those relative shifts.

PACS No. 76.50.+q; 84.37.+q; 75.70.-i

## Introduction

It is known that impedance (IM) of an ferromagnetic (FM) material is closely related to its complex permeability (*μ *≡ *μ*_R _+ *i μ*_I _), where *μ*_R _and *μ*_I _are the real and imaginary parts, in the high-frequency (*f*) range [1,2]. Past experience has also shown that there should exist a cutoff frequency (*f*_c_), where *μ*_R _crosses zero and *μ*_I _reaches maximum [3], for each FM material. According to Ref. [3], *f*_c _increases as the thickness of the FM sample decreases and finally reaches an upper limit. The thickness dependence is due to the eddy current effect, while the upper limit is due to the spin relaxation (or resonance) effect. Hence, in a sense, we would expect the *f *dependence of impedance *Z *= *R *+ *iX*, where *R *is resistance and *X *reactance, behaves similarly. In Ref. [1], we had the situation that the thickness (*t*_F_) of the FM ribbon was thick to meet the criterion: *t*_F _≥ *δ*≅ 10 μm, where *δ*is skin depth (at *f *= 1 MHz), but in this article, we have a different situation wherein the thickness (*t*_f_) of the FM film is thin to meet the criterion: *t*_f _= 100 nm <<*δ*≅ 654 nm (at *f *= 1 GHz). That means the time varying field *H*_g_, generated by the ac current (*i*_ac_), in the IM experiment should penetrate through the film sample even under an ultrahigh frequency condition this time. Moreover, there are various kinds of mechanisms to explain the resonance phenomena: the film size (FZ), the magnetic domain wall (MDW), the RLC-circuit, the ferromagnetic resonance of the Kittel mode (FMR-K), the ferromagnetic resonance of the Walker mode (FMR-W), the relaxation time, and the standing spin-wave resonance mechanisms. We shall examine all these mechanisms one by one, based on the experimental data collected in this study.

## Experimental

**Figure 1:**
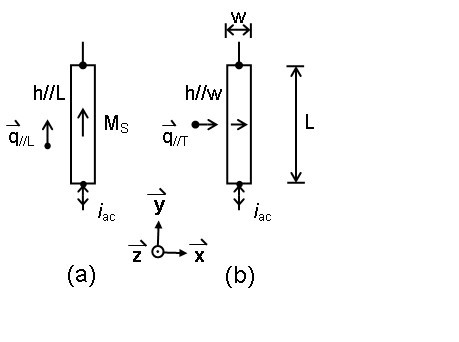
**Two Co_40_Fe_40_B_20 _film samples**. *L *is the length and *w *the width. *i*_ac _is the ac current sent through each sample.  is the saturation magnetization and  is the deposition field.  and  are the in-plane spin-wave wave vectors. **(a) **The ||*L *case and **(b) **the ||*w *case.

The composition of the film sample in this test was Co_40_Fe_40_B_20_. We used magnetron sputtering technique to deposit the film on a glass substrate at room temperature. The film thickness *t*_f_, as mentioned before, was 100 nm. During the deposition period, an external dc field, *h *≅ 40 Oe, was applied to define the easy axis, as shown in Figure 1, for each nanometer thick sample. In Figure 1, we have length, *L *= 10.0 mm, and width, *w *= 500 μm, in case (a) *h*||*L*, and in case (b) *h*||*w*.  is the saturation magnetization of each film. In addition, the nanocrystalline grain structures in our CoFeB films were confirmed from their transmission electron microscope photos.

In a typical IM experiment, there were three features: (1) the rectangular film sample, either as shown in Figure 1a or Figure 1b, was placed at the center of a pair of Helmholtz coils, which could produce a field *H*_E _⊥ *L*, (2) *Z *was measured by an Agilent E4991A RF impedance/material analyzer (Agilent Technologies, Santa Clara, CA, USA) with a two-point (ECP18-SG-1500-DP) pico probe, and (3) the peak-to-peak amplitude of the ac current, *i*_ac_, was fixed at 10 mA, and the frequency *f *of the current was scanned from 1 MHz to 3 GHz.

**Figure 2:**
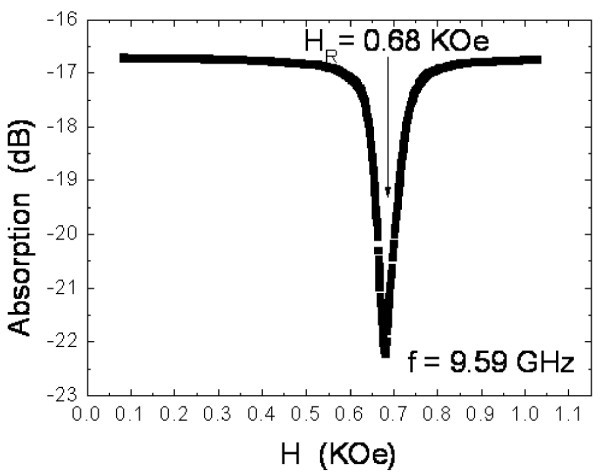
**Ferromagnetic resonance of the Co_40_Fe_40_B_20 _film with the microwave frequency *f *= 9.6 GHz**. *H*_R _is the resonance field.

A circular film sample was taken for the FMR experiment. The cavity used was a Bruker ER41025ST X-band resonator (Bruker Optics Taiwan Ltd., San Chung, Taiwan, Republic of China) which was tuned at *f *= 9.6 GHz, and the film sample was oriented such that ||and  ⊥ , where  was an in-plane field which varied from 0 to 5 kG, and  was the microwave field. The result is shown in Figure 2, where we can spot an FMR (or FMR-K) event at *H *= *H*_R _= 0.68 kG, and define the half-peak width Δ*H *= 53 Oe.

Other magnetic and electrical properties of the Co_40_Fe_40_B_20 _film were obtained from vibration sample magnetometer measurements: 4*πM*_s _= 15.5 kG and the anisotropy field, *H*_k _= 0.031 kG, and from electrical resistivity (*ρ*) measurement: *ρ *= 168 μΩ. cm. Note that because of the nanocrystalline and the nanometer thickness characteristics, the *ρ *of our Co_40_Fe_40_B_20 _films is very high. Here, since δ ∝ (*ρ*)^1/2^, a larger ρ will lead to a longer δ >>*t*_f_.

## Results and discussion

**Figure 3:**
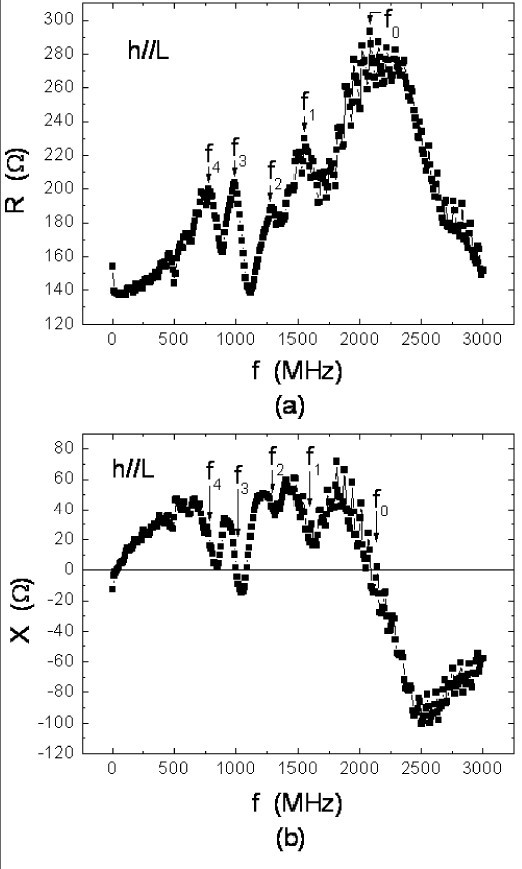
**Impedance *Z *= *R *+ *iX *with  || L**. Impedance *Z *= *R *+ *iX *where *R *and *X *are the resistance and reactance of the Co_40_Fe_40_B_20 _film sample with ||*L*. *f*_0 _and *f_n_*, with *n *= 1, 2, 3, 4, are the frequency peaks associated with various kinds of resonances.

**Figure 4:**
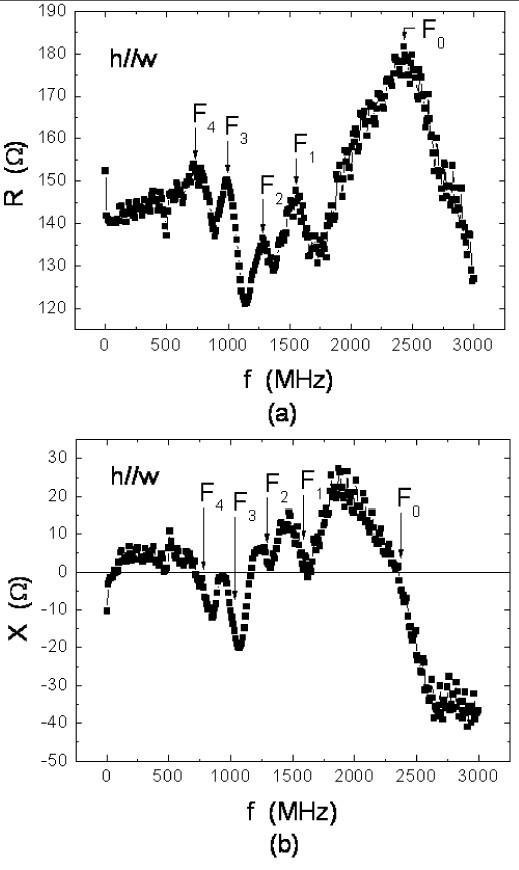
**Impedance *Z *= *R *+ *iX *but with ||*w***. *F*_0 _and *F_n_*, with *n *= 1, 2, 3, 4 are the frequency peaks associated with various kinds of resonances.

In order to interpret the IM data (or spectrum) of this work, as shown in Figure 3 (the *h*||*L *case) and in Figure 4 (the *h*||*w *case), we have the following definitions. First, whenever there is a resonance event, we should find a peak located at *f *= *f*_0 _and *f *= *f_n_*, where *n *= 1, 2, 3, 4 in the R-spectrum, and a wiggle (or oscillation) centered around the same *f*_0 _and *f_n _*in the X-spectrum. To summarize the data in Figures 3 and 4, we have in the *h*||*L *case, *f*_0 _= 2,081, *f*_1 _= 1,551, *f*_2 _= 1,291, *f*_3 _= 991, and *f*_4 _= 781 MHz; and in the *h*||*w *case, *F*_0 _= 2,431, *F*_1 _= 1,551, *F*_2 _= 1,281, *F*_3 _= 991, and F_4 _= 721 MHz. From these experimental facts, we reach two conclusions: (1) *f*_0 _≠ *F*_0 _and (2) within errors, *f_n _*= *F_n_*. Since at either *f*_0 _or *F*_0_, each corresponding wiggle crosses zero, we believe there is a quasi-FMR-K event. Notice for the moment that because *f*_0 _≠ *F*_0_, we use the prefix "quasi" to describe the event. More explanation will be given later.

Here, we discuss the possibilities of the FZ resonance first. From Ref. [4], we know an electromagnetic (EM) wave may be built up inside the film during IM experiments. In Figure 1a, supposing *L *≅ *λ*_||_, where *λ*_|| _is the longitudinal EM wavelength, *w *≅ *λ*_⊥_, where *λ*_⊥_is the transverse EM wavelength, and *μ *≅ 10^3^, we find the FZ resonance frequencies: *f*_EM_(||) = *η*_|| _× 7 MHz and *f*_EM_(⊥) = *η*_⊥ _× 27 MHz, where *η*_|| _and *η*_⊥_are positive integers. Since based on the experimental findings, *f_n _*= *f*_EM_(||) should be equal to *F_n _*= *f*_EM_(⊥), *f_n _*or *F_n _*must be a positive integer number of times of the frequency 189 MHz. Simple calculations show that the above statement cannot be satisfied. Besides, if the statement were true, there would exist at least as many as eight different FZ resonance peaks, instead of only the four resonance peaks observed so far.

Next, the MDW mechanism is discussed. As the size of the sample is large, there are magnetic stripe domains, parallel to  in Figures 1a, b. According to Ref. [5], the MDW resonance for the CoFeB film should occur at *f *= 78 MHz. However, we have reasons to believe that this kind of resonance does not exist in our IM spectra. First, in Figures 3 and 4, there is neither a peak nor a wiggle at *f *= 78 MHz. Second, when *H*_E _= 150 G, much larger than the saturation field, was applied to eliminate magnetic domains, those peaks (at *f*_0 _to *f*_4 _or *F*_0 _to *F*_4_, respectively) still persisted.

Further, the RLC-circuit resonance mechanism is discussed. If the Co_40_Fe_40_B_20 _film is replaced by a Cu film with the same dimensions, there is also one single resonance peak at *f*_d_(Cu) = (1/2*π*)(*L*_s_*C*)^-(1/2) ^= 2.641 GHz, where *L*_s _is the self-inductance and *C *is the capacitance of the film [6]. However, we believe that *f*_0 _and/or *F*_0 _are less likely due to the RLC-circuit resonance mechanism for the reason below. Since *L*_s _= μ × GF ~(10^2 ^to 10^3^) × *μ*_o _× GF for Co_40_Fe_40_B_20_, where GF depends only on the geometrical size and shape of the sample, *L*_s _= 1 × *μ*_o _× GF for Cu, and *C*_CoFeB _≥ *C*_Cu_, in principle, we find *f*_d_(Co_40_Fe_40_B_20_) ≅ [(1/10) to (1/30)] × *f*_d_(Cu) = 0.26 to 0.08 GHz, which is too small to meet the facts, i.e., *f*_0 _= 2.081 GHz and *F*_0 _= 2.431 GHz.

With regard to the FMR-W mechanism, we have the following discussion. At *f *= *f_n _*and/or *F_n_*, we believe each resonance should correspond to a specific FMR-W mode. The reasons are summarized below. First, in the typical FMR result, as shown in Figure 2 because the sample was placed in the homogeneous *h*_rf _region, no FMR-W modes could be observed. However, as indicated in Ref. [4], if *h*_rf _is sufficiently inhomogeneous to vary over the sample, one will observe various FMR-W modes at *H *= *H_n _*and *H_n _*<*H*_R_. From a simple relationship [4], such as *f *= *νH*_eff_, where *H*_eff _is the effective field and ν = *γ*/2*π *is the gyromagnetic ratio, it is easy to recognize that since *H_n _*<*H*_R_, we have *f_n _*<*f*_0 _and/or *F_n _*<*F*_0_, which is what has been observed. Second, from Refs. [7] and [8], it is known that *h*_rf _≡ *H*_g _= (*i*_ac_*z*)/(w*t*_f_), where *z *is a variable parameter along *t*_f_. Therefore, in a typical IM measurement, *h*_rf _or *H*_g _cannot be homogeneous all over the sample. That is why in Figure 2, there is no FMR-W mode, but in Figures 3 or 4, there are various FMR-W modes.

With regard to the FMR-K mechanism, we propose the following model: When FMR-K occurs in Figure 2, we have [9].(1)

By substituting the values of *f*_R _= 9.6 GHz, *H*_R_, *H*_K_, and 4*πM*_s_, it is found ν = 2.833 for Co_40_Fe_40_B_20_. Thus, the main (or FMR-K) resonance (at *H *= 0) would occur at *f *= *f*_FMRK _= ν[*H*_K_(*H*_K _+ 4*πM*_s_)]^1/2 ^= 1,963 MHz. According to our previous arguments, this frequency, *f*_FMRK_, should be equal to *f*_0 _and/or *F*_0 _in MI. Obviously, what we have is *f*_FMRK _≠ *f*_0 _≠ *F*_0_. The reasons for the frequency shifts of the quasi-FMR-K resonances in IM are given below. According to Refs. [9-11], the quasi-FMR-K-resonance relationship for *f*_0 _or *F*_0 _at *H *= 0 and under the exchange-dominated condition is expressed as(2)

where *A *= 1.0 × 10^-11 ^J/m is the exchange stiffness, *i *= *L *or *T*, *q*_//*i *_is the in-plane (IP) standing spin-wave wavevector, (*pπ*/*t*_f_) is the out-of-plane (OFP) standing spin-wave wavevector, *p *= 0, 1, 2,...etc., *θ_q _*is the angle between  and the surface normal  or the *z*-axis, hence for  and , as shown in Figure 1, *θ_q _*= *π*/2 always, and *τ *is the relaxation time [9], where 1/*τ *≡ (*αγH*_R_) = 94.3 MHz and *α *≡ ν(Δ*H*)/(2*f*_R_) = 0.00777. Therefore, if the relaxation time (1/τ) mechanism dominated in Equation 2, *f*_0 _would be equal to 267 MHz, which is much lower than the *f*_0 _or *F*_0 _in Figures 3 and 4.

Next, we consider the OFP standing spin-wave case only, i.e., temporarily assuming *q*_//*i *_= 0 or negligible in Equation 2, simple calculations show that *f*_0_(*p *= 0) = 1.963 GHz, *f*_0_(*p *= 1) = 4.874 GHz, and *f*_0_(*p *= 2) = 9.136 GHz. Because our Agilent E4991A works only up to 3.0 GHz, *f*_0_(*p *= 1) and *f*_0_(*p *= 2), although existing, were not observed in this work.

In the following, we shall refer to the *p *= 0 case only. From Equation 2, if *p *= 0 and the (1/*τ*) term is negligible, we consider the following two cases: in Figure 1a, ||*L*, where the azimuth angle *φ *of  is (*π*/2) and in Figure 1b, ||*w*, where *φ *= 0. Then, Equation 2 can be simplified as(3a)(3b)

By substituting the values of *f*_0_, *F*_0_, *A*, and *H*_k _in Equations 3a, b, respectively, we find *q*_//*L *_= 1.326 × 10^6 ^(1/*m*) and *q*_//*T *_= 3.216 × 10^6 ^(1/*m*). Two features can be summarized. First, since [1/(2π)][*q*_//*i *_× *t*_f_] = (0.5 to 1.2) × 10^-1 ^<< 1, it confirms that we do have a long wavelength in-plane spin wave (IPSW), *q*_//*L *_or *q*_//*T*_, traveling in each film sample. Second, due to the boundary conditions of the film sample, we should have *q*_//*L *_∝ (1/*L*) and *q*_//*T *_∝ (1/*w*). Thus, because *L *>*w*, our previous results are reasonable that *q*_//*L *_<*q*_//*T*_.

Finally, as to why the IP spin-waves can be easily excited in the IM experiment, but cannot be found in the FMR experiment, we have a simple, yet still incomplete, explanation as follows. The film sample used in the latter experiment is circular, which means by symmetry *L *= *w*, while the one used in the former experiment is rectangular, which means that the symmetry is broken, with *L *≠ *w*. Thus, even if  exists in the FMR case, there should be only one , where , by symmetry argument. Nevertheless, for some reasons, such as (1) that a high-current density *j*_ac _= (*i*_ac_)/(*t*_f_*w*) may be required to initiate IPSW, and (2) that *j*_ac _flowing in the FMR experiment may be too low to initiate any IPSW, we think the *q*_//_term in  is likely to be negligible. As a result, in Figure 2, we find only one  in the FMR case and  = *f*_FMRK_. However, due to reason (1) above, and the symmetry breaking issue in the IM case, as discussed before,  should be shifted from *f*_FMRK _to *f*_0 _and *F*_0_, respectively.

**Figure 5:**
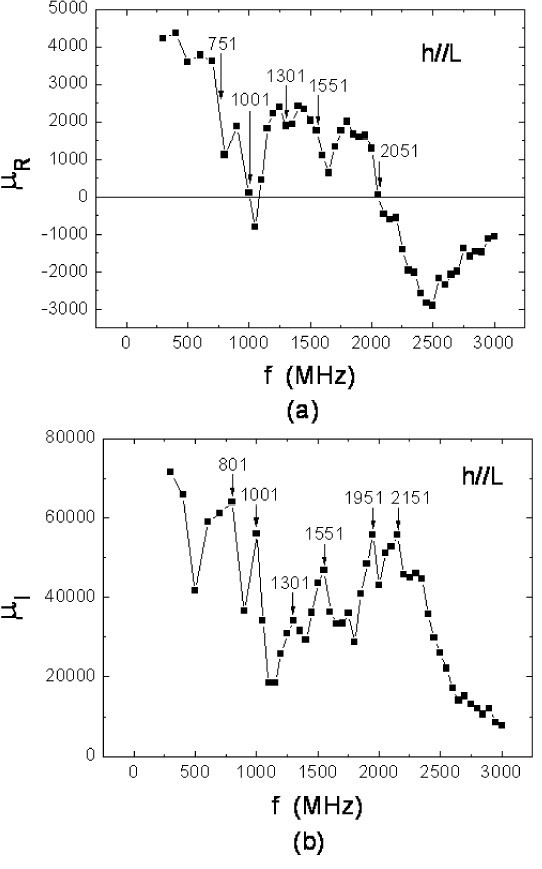
**Permeability μ = μ_R _+ *i μ*_I_**. Permeability μ = μ_R _+ *iμ*_I _where μ_R _and μ_I _are the real and imaginary parts of the film samples vs. the frequency *f*.

Moreover, if we take the formula *Z *= (*B*/*A*_s_)(1 + *i*)coth[(*t*/2*A*_s_)(1 + *i*)], where *B *= (*ρL*)/(2*w*), *A*_s _= [*ρ*/(*πfξμ*_o_)][cos(*δ*/2) + *i*sin(*δ*/2)], *μ *≡ *ξμ*_o_, and *μ*_o _= 4*π *× 10^-7 ^H/m. By using the Newton-Raphson method [12], we may calculate the *f *dependence of *μ*_R _≡ ξcos*δ *or *μ*_I _≡ -ξsin*δ *from the *R *and *X *data. From the *μ*_R _vs. *f *or the *μ*_I _vs. *f *plot, as shown in Figure 5, we can define the cutoff frequency *f*_c _= 2,051 MHz in the *h*||*L *case. Clearly, *f*_c _in Figure 5 is almost equal to *f*_0 _found in Figure 3.

## Conclusion

We have performed IM and FMR experiments on nanometer thickness Co_40_Fe_40_B_20 _film samples. Film thickness *t*_f _was deliberately chosen much smaller than eddy current depth δ in the frequency range 100 MHz to 3 GHz. From the FMR data, we find that the Kittel mode resonance occurs at *f*_FMRK _= 1,963 MHz, while from the IM data, we find that (1) the quasi-Kittel-mode resonance occurs at *f*_0 _= 2,081 MHz in the *h*||*L *case and *F*_0 _= 2,431 MHz in the *h*||*w *case, respectively, and (2) the Walker-mode resonances at *f_n _*= *F_n _*for both cases. It is believed that the shift of  from *f*_FMRK _to *f*_0 _or from *f*_FMRK _to *F*_0 _is due to the existence of IPSWs. Moreover, we have estimated the values of wave vectors of IPSW,  in the *h*||*L *case and  in the *h*||*w *case, and found that  is smaller than  as expected.

## Competing interests

The authors declare that they have no competing interests.

## Authors' contributions

SUJ designed and directed this research, analyzed and interpreted the data, and wrote the paper.

TYC carried out the IM and FMR experiments.

CKL set up and provided the FMR equipments.
